# CRIMSON [CRisis plan IMpact: Subjective and Objective coercion and eNgagement] Protocol: A randomised controlled trial of joint crisis plans to reduce compulsory treatment of people with psychosis

**DOI:** 10.1186/1745-6215-11-102

**Published:** 2010-11-05

**Authors:** Graham Thornicroft, Simone Farrelly, Max Birchwood, Max Marshall, George Szmukler, Waquas Waheed, Sarah Byford, Graham Dunn, Claire Henderson, Helen Lester, Morven Leese, Diana Rose, Kim Sutherby

**Affiliations:** 1 Health Service and Population Research Department, Institute of Psychiatry, King's College London, UK; 2 Department of Psychology, University of Birmingham, Birmingham, UK; 3 Division of Psychiatry, School of Medicine, University of Manchester, Manchester, UK; 4 Community Based Medicine, University of Manchester, Manchester, UK; 5 National School of Primary Care, The University of Manchester, Manchester, UK

## Abstract

**Background:**

The use of compulsory treatment under the Mental Health Act (MHA) has continued to rise in the UK and in other countries. The Joint Crisis Plan (JCP) is a statement of service users' wishes for treatment in the event of a future mental health crisis. It is developed with the clinical team and an independent facilitator. A recent pilot RCT showed a reduction in the use of the MHA amongst service users with a JCP. The JCP is the only intervention that has been shown to reduce compulsory treatment in this way. The CRIMSON trial aims to determine if JCPs, compared with treatment as usual, are effective in reducing the use of the MHA in a range of treatment settings across the UK.

**Methods/Design:**

This is a 3 centre, individual-level, single-blind, randomised controlled trial of the JCP compared with treatment as usual for people with a history of relapsing psychotic illness in Birmingham, London and Lancashire/Manchester. 540 service users will be recruited across the three sites. Eligible service users will be adults with a diagnosis of a psychotic disorder (including bipolar disorder), treated in the community under the Care Programme Approach with at least one admission to a psychiatric inpatient ward in the previous two years. Current inpatients and those subject to a community treatment order will be excluded to avoid any potential perceived pressure to participate. Research assessments will be conducted at baseline and 18 months. Following the baseline assessment, eligible service users will be randomly allocated to either develop a Joint Crisis Plan or continue with treatment as usual. Outcome will be assessed at 18 months with assessors blind to treatment allocation. The primary outcome is the proportion of service users treated or otherwise detained under an order of the Mental Health Act (MHA) during the follow-up period, compared across randomisation groups. Secondary outcomes include overall costs, service user engagement, perceived coercion and therapeutic relationships. Sub-analyses will explore the effectiveness of the JCP in reducing use of the MHA specifically for Black Caribbean and Black African service users (combined). Qualitative investigations with staff and service users will explore the acceptability of the JCPs.

**Discussion:**

JCPs offer a potential solution to the rise of compulsory treatment for individuals with psychotic disorders and, if shown to be effective in this trial, they are likely to be of interest to mental health service providers worldwide.

**Trial registration:**

Current Controlled Trials ISRCTN11501328

## Background

Two of the key guiding principles of mental health policy in England are that service users should: (i) exercise choice and control over their treatment [1], and (ii) be subjected to the least restrictive form of care. Service users now routinely participate in care planning, service development and research. Nevertheless, against the European trend [2], the use of compulsory treatment in England has continued to rise [3]. This conflicts with government policy and service user preferences given that many service users find in-patient wards counter-therapeutic [4].

In England, in addition to the general concern about rising rates of compulsory treatment, there is additional concern that Black African and Black Caribbean service users experience more coercive mental health care than their white counterparts [5-8]. So far no interventions have been identified which reduce compulsory mental health treatment for Black Caribbean and Black African service users.

A Joint Crisis Plan (JCP) aims to empower service users whilst facilitating early detection and treatment of relapse [9,10]. It is developed by the mental health service user in collaboration with staff. Held by the service user, it contains his or her treatment preferences for any future psychiatric emergency, when he or she may be too unwell to express clear views. The JCP format has developed over the last decade after widespread consultation with national service user groups, interviews with organisations and individuals using JCPs, and detailed developmental work with service users in South London [9,10]. Initial findings from the pilot study suggest that the negotiation process itself helps engage the service user and the clinician in a more acceptable, collaborative, and beneficial therapeutic relationship.

JCPs are somewhat similar to Psychiatric Advance Directives (PAD) and Facilitated Advance Directives (F-PADs) that have been developed in the United States (see [11]). Non-Randomised studies from the United States suggest that PADs are popular with service users [12,13] and generate clinically relevant information [14]. From a randomised trial, there is also preliminary evidence to suggest that F-PADs may improve therapeutic relationships between service users and clinicians [15] and reduce coercive crisis interventions such as police transport, forced medication, use of seclusion and restraints, and involuntary commitment [16]. However, JCPs differ from the F-PADs and PADs in two important ways: firstly, JCPs require the direct involvement of the clinical team in helping the service user decide the content of the JCPs; and secondly JCPs do not have the same degree of medico-legal enforceability [11]; advance refusals of treatment must be respected under the terms of the Mental Capacity Act 2005 but can be over-ridden by use of an involuntary treatment order under the Mental Health Act (1983).

Two studies have produced findings that suggest that JCPs might reduce compulsory treatment and improvement therapeutic relationships. A pilot study of JCPs conducted by the authors in South London [9] showed that at 6 to 12 month follow-up, 57% of participating psychiatric service users with JCPs reported feeling more involved in their care, 60% were positive about their situation, 51% felt more in control of their mental health problem and 41% felt they were more likely to continue treatment. Further, an exploratory randomised study of JCPs [17], found that use of the MHA was significantly reduced for the intervention group, 10/80 (12.5%) of whom experienced compulsion versus 21/80 (26.5%) of the control group (risk ratio 0.48, 95% CI 0.24 to 0.95, p = 0.028). While overall bed-day use was not significantly different between experimental and control groups, the mean number of days compulsorily detained under the Mental Health Act (1983) for the intervention group during the 15 month follow-up period was 14 compared to 31 for the control group (difference 16, 95% CI 0 to 36, p = 0.04). In summary, coercive treatment in the form of MHA use was halved by the use of JCPs: the first structured clinical intervention shown to reduce compulsion in mental health services. JCPs are relatively straightforward to implement, and offer the prospect of less restrictive care for service users with psychotic disorders. A more recent health economic analysis of this trial has shown that the intervention has a highly probability of being more cost-effective than the control [18]. The time is therefore right for a definitive trial.

The primary hypothesis to be tested is whether JCPs significantly reduce the proportion of service users detained or treated under a section of the MHA during the 18 month follow-up period, compared with the control group. Secondary hypotheses will be to determine if compared with the control condition, JCP use will result in significant improvements in: total costs, perceived coercion, service user engagement, therapeutic alliance. Sub-analyses will examine the effectiveness of the JCP in reducing use of the Mental Health Act for Black (Black Caribbean and Black African) service users.

## Methods/Design

This trial is funded by the Medical Research Council (MRC). This trial has received ethical approval from King's College Hospital Research Ethics Committee.

### Design

The CRIMSON trial is an individual-level single-blind Randomised Controlled Trial of Joint Crisis Plans (JCPs) compared with a treatment as usual control for people with a history of relapsing psychotic illness under the care of community mental health teams in Birmingham, London and Lancashire/Manchester. The total duration of the study will be four years, to allow for: recruitment to target; provision of the intervention; follow-up assessments; qualitative exploration of use and acceptability of the JCPs; and data analysis, using intention to treat methods. Recruitment to the trial began in August 2008 and is due for completion in March 2010. Follow-up assessments begin in February 2010 and will be completed by September 2011.

#### The intervention

The Joint Crisis Plan (JCP) intervention has been described in some detail elsewhere [9-11], but is briefly delineated here. At each site, a trained and clinically experienced Facilitator will organise two meetings with each service user randomised to receive a JCP. In the first meeting, the Facilitator will introduce the JCP 'menu' (a list of topics to be considered for inclusion in the JCP) to each service user. This first meeting will occur in either the service user's home or a local clinical setting. The facilitator will then organise a subsequent planning meeting between the service user, team consultant psychiatrist or other psychiatrist, and the Care Co-ordinator, when the JCP contents will be finalised. This planning meeting will be convened, usually at the clinical base, a minimum of one week after the preliminary meeting to enable the service user time to consider their options. The service user is encouraged to bring a carer or friend to act as an advocate to the planning meeting. The JCP contains: information on early warning signs of relapse and advance treatment statements; contact details of primary and secondary care staff for routine and emergency care; details of medication; psychiatric and physical diagnoses; allergies; and details of who will have a copy of the JCP. A copy of the JCP is included on the computerised patient information systems in routine use by the clinical services. In addition, the Facilitator provides a printed version of the JCP to all those specified by the service user.

To ensure fidelity of the intervention at each site:

i) Facilitators will be experienced mental health professionals who will receive rigorous training in negotiating joint crisis planning meetings, and producing JCPs;

ii) continual supervision by one of the developers of the intervention (KS) will be provided;

iii) fidelity to the model will be formally assessed at random intervals using a rating scale to evaluate audiotapes of meetings and copies of completed JCPs;

i) issues identified during supervision or fidelity assessment will be ivmmediately addressed through further training and supervision.

#### The control condition

We have chosen to use a treatment as usual (TAU) control condition, as this provides a fair comparison with routine clinical practice, and will answer the question of whether JCP use is superior to current standard care. TAU includes, as a part of the Care Programme Approach (CPA), the need for service users to receive written copies of their care plan [19] including a 'crisis contingency plan', as indeed was the case in our recent exploratory trial. We expect that the CPA arrangements, and the new 2006 Quality and Outcomes Framework (QOF) (which requires a care plan to be documented in primary care case records), will be applied equally to intervention and control groups. The content of the Care Programme Approach of all participants will be assessed at baseline and follow-up.

### Participants

#### Inclusion and Exclusion Criteria

Eligible service users will be adults (age 16+) and will have: (i) contact with a local Community Mental Health Team (CMHT) (including assertive outreach teams, early intervention teams, and community forensic teams, but not home treatment teams.); (ii) been admitted to a psychiatric in-patient service at least once in the previous two years; (iii) a diagnosis of psychotic illness, including bipolar affective disorder (using Operational Criteria Checklist OPCRIT [20]), and (iv) been on the local NHS Trust Enhanced CPA/CPA Register in the last two years. We shall include service users who do not speak English. For non-English speakers, both written translation and interpreters are needed and we shall employ interpreters as required.

We will exclude participants unable to give informed consent. Additionally, current in-patients and those subject to a compulsory community treatment order will not be recruited to avoid any perceived coercion to participate. No other exclusions will be made to maximise the external validity of the trial.

### Recruitment and Randomisation

A list of eligible service users will be generated by Clinical Studies Officers from the Mental Health Research Network and maintained by the Study Co-ordinating Centre. Each eligible person, with the agreement of the clinical team, will be approached by a trained member of the research team and invited to participate in the study, thus eliminating selection biases. Once informed consent has been obtained, trained researchers will conduct a baseline assessment and then follow-up each participant 18 months later. The setting for the research assessments will be the choice of the participant.

After the baseline assessment, participants will be randomly allocated to either intervention or control group, stratified by centre using permuted blocks of randomly varying block size, with equal allocation to the two arms. To ensure concealment of allocation the randomisation will be performed by an online system managed by the Clinical Trials Unit at the Study Coordinating Centre in London.

### Assessments

At baseline and follow-up, service user socio-demographic information (including education, employment, clinical diagnoses and marital status) will be collected. Overall functioning will be measured by Global Assessment of Functioning (GAF) at baseline and follow-up assessments. Information will be obtained from interviews with participants and case notes with the service user's permission. Additionally, some demographic information will be collected on the service user's care coordinator such as age, qualifications and length of practice. All data will be collected by research assistants blind to allocation. Maintenance of blinding will be recorded and reported at the end of the trial.

#### Primary Outcome Measure

The primary outcome is the proportion of service users treated or otherwise detained under an order of the Mental Health Act (MHA) during the follow-up period. MHA data will be gathered from and validated between the following sources: case notes, the local Patient Administration System, Mental Health Act Office data, and interviews with service users and Care Co-ordinators (the latter being particularly important to pick up out of area admissions). A sub-analysis of this data will examine the proportion of Black service users treated or detained under the MHA.

#### Secondary Outcome Measures

The secondary outcomes include mental hospital use data, total costs, perceived coercion, engagement and therapeutic relationships.

##### Hospital use data

The proportion of service users entering hospital voluntarily, the number of days spent in hospital both voluntarily and under section, and time to first admission will be compared between groups. This data will be collected with the primary outcome data.

##### Costs

The economic evaluation will take a broad perspective and will be based on comprehensive resource use data collected on all health, social care, housing and other community support services used by individual trial participants, contact with criminal justice agencies, and other resources arising from the use of the MHA, and productivity losses. Data will be collected in interviews with participants at baseline and follow-up using the Adult Service Use Schedule (AD-SUS), a measure designed by the applicants (SB) for collecting service use data in mental health populations. Interview data will be supplemented by data on hospital contacts, the key cost driver in this population, collected from routine computerised hospital records. Data on the time and staffing required in undertaking the JCP intervention will be collected from JCP facilitator records, as in our previous trial [17].

##### Perceived Coercion

Perceived coercion will be measured using an adapted version of the MacArthur Perceived Coercion Scale. This scale has been adapted for reference to outpatient treatment [21] and is designed to ascertain participants' experience of coercion during treatment in the community. This is a self-report measure completed by the service user at baseline and follow-up. Responses to this measure will be used to generate three scales: 'perceived coercion', 'negative pressures' and 'process exclusion'.

##### Engagement

Two measures of engagement will be completed by the care coordinator at baseline and follow-up. The first measure is adapted from the Homeless Engagement and Acceptance Scale [22]. The full scale has five items. The adapted version drops the last item that refers to homelessness. The second measure of Engagement is the Service Engagement Scale [23]. This is a 14 items scale producing four subscales measuring 'availability', 'collaboration', 'help seeking' and 'treatment adherence'. Both measures of engagement are rated by the participant's care coordinator.

##### Therapeutic relationship

Both service users and staff will complete the short version of the Working Alliance Inventory adapted for use for individuals with severe mental illness [15,24,25].

##### Recovery Style

We will also examine participant recovery styles with the Recovery Style Questionnaire (RSQ) [26]. The RSQ has 39-items which are rated by the service user. The RSQ is used to classify four recovery styles: 'Integration'; 'Mixed picture in which integration predominates'; 'Mixed picture in which sealing over predominates'; and 'Sealing over'.

#### Qualitative Analyses

Qualitative methods will be carried out to explore the processes through which JCPs achieve change in practice. Pilot work suggests they have direct and indirect effects. Negotiating JCP content may clarify treatment issues and build consensus between service users and staff. However other important effects may include changes in: trust; service user engagement in the process of care including shared decision making [27]; service user self esteem and empowerment; channels of communication between all parties; staff risk perception; and/or changes within the culture of the mental health service may also be important. Focus groups will be used to examine people's experience of the JCP. The important 'break characteristic' used in data analysis will be whether service users with a JCP were subject to MHA use. 18 focus groups (6 groups in each site) will run 18-21 months after entry to the study.

We will conduct separate service user and health professional focus groups with service users who have or have not been treated under the Mental Health Act. These groups will be followed, usually one week later, by a combined subset of the service users and health professionals who attended the separate groups. We expect to involve 16 service users in each geographical site and a similar number of professionals involved in the development of the JCP with the service user. Focus group topic guides will be developed from a literature review and include the themes discussed above. Combined groups will additionally explore the roles and responsibilities of service users and health care professionals in relation to the use of the JCP and the influence on and impact of the culture of mental health services.

To understand the experiences and views of psychiatrists who have participated in the intervention, a series of individual interviews will be conducted. Interviews will cover the psychiatrist's views on the process of the intervention, the outcome and the impact of such an intervention on mental health services. Interviews will be conducted in all sites, and will continue until 'data saturation' is achieved i.e., no further themes are identified.

Focus groups and psychiatrist interviews will be audio taped and fully transcribed. Transcripts and notes will be read and re-read independently by two of the research team. The data will then be organised into initial codes and higher codes that provide insight into emergent themes. For the focus groups, reliability will be enhanced by identifying issues that are consistent between groups and validated using so-called 'sensitive moments' within group interactions that indicate difficult but important issues. A computer software package will be used to manage the data and increase the transparency of the analysis. Deviant cases will be actively sought throughout the analysis and emerging ideas and themes modified in response.

### Analysis

#### Power calculation

The primary outcome is reduction in the proportion of service users treated or detained under a Mental Health Act section at least once over the 18 month follow-up period. In the pilot trial, based in London, 26% were compulsorily admitted over 15 months, equivalent to 30% over 18 months. Routine data for inner city wards in Birmingham and Manchester show a very similar proportion, on average. Assuming that a clinically important reduction would be to at least halve the proportion, i.e. a reduction in absolute terms by 15% to 15%, 90% power using a double-sided test with alpha = 0.05 would require 174 in each arm. For the ethnic subgroup analysis where the baseline compulsory admission rate is expected to be higher, an achieved subsample of 91 per arm would give 80% power to detect a difference from 40% to 20%. Given the percentages of service users likely to be black at each site (from recent MHA use data), 90 are likely to be found with a sample of 270 per arm, and the minimum achieved would be about 80 with a slight reduction in power. Loss to follow-up is likely to be about 15% for the interview data so this sample size (270) would reduce to an effective 229 per arm, which would allow standardised effect sizes of 0.3 for the secondary outcomes to be detectable with 90% power. The total to be recruited would therefore be 540, or 180 per site

#### Analysis Plan

The principal analysis of effectiveness will compare the primary and secondary outcome measures at 18 months, combined over centres. The proportions admitted to hospital under a section at follow up will be compared between randomisation groups using logistic regression controlling for centre. Other (continuous) outcomes such as therapeutic alliance and engagement with mental health services will be analysed using analysis of covariance controlling for baseline (pre-intervention) measures and centre. Number of admissions will be analysed using Poisson regression, and time to first admission using survival analysis. Bed-days and other very skewed data will be analysed using bootstrapping to obtain confidence intervals and p-values [28]. Short scales will be analysed using ordered logistic regression. An intention-to-treat analysis will be applied in the first instance (i.e. analysing all available data from service users as randomised). Time trends in measures available at baseline and two time points will be analysed using methods for longitudinal data such as random effects regression. Sensitivity analyses will be performed to assess the influence of loss to follow-up and refusals, including imputation of missing baseline values from the within-centre means; multiple imputation of follow up values (where feasible from other variables) and CACE analyses [29].

#### Health Economic analysis plan

The JCP intervention will be costed using recognised micro-costing methods [30]. Costs will be based on the mid-point of the salary scale of the relevant JCP professionals, including all employer costs (National Insurance and Superannuation contributions) and appropriate overhead costs (capital, administration, managerial etc). Other services will be costed using locally applicable estimates of long-run marginal opportunity costs. Where necessary, these will be supplemented by published national unit costs [31]. Nationally applicable unit costs will be applied in sensitivity analysis to assess the generalisability of the cost results to the UK as a whole. Productivity losses will be costed using the human capital approach, which involves multiplying days off work due to illness by the individual's salary level [32]. A number of commentators have argued that this approach is limited since it tends to overestimate productivity losses by ignoring, for example, the ability to replace workers from the pool of unemployed people [33]. Given these limitations, the impact of varying productivity losses will be explored in sensitivity analysis.

The mean costs in the two groups will be compared with confidence intervals for mean differences estimated using bootstrapping controlling for centre and baseline costs. Future costs and outcomes will not be discounted in the main analyses because the single follow-up point will not allow costs that fall within the first 12 months to be separated from those which occur subsequently. However, the potential impact of excluding discounting will be explored in sensitivity analyses. Cost-effectiveness will be explored using the proportion admitted under a section over the 18-month follow-up period. Incremental cost-effectiveness ratios (of the additional costs to the additional effects of the JCP process in comparison to the control group) [34] will be reported and cost-effectiveness acceptability curves showing the probability that the JCP is more cost-effective than the control condition will be plotted [35]. Supplementary evaluation will take the form of a cost-consequences analysis examining total costs in relation to all other secondary outcomes. SPSS version 12 and Stata version 9 will be used for the analyses.

## Discussion

Previous data shows that the Joint Crisis Plan intervention could halve compulsory treatment under the Mental Health Act and has a high probability of being cost effective. The Joint Crisis Plan intervention therefore offers a potential solution to the rise of compulsory treatment for individuals with psychotic disorders. If shown to be effective in this trial, it is likely Joint Crisis Plans will have wide implications for practice and involving service users in their care.

## Competing interests

The authors declare that they have no competing interests.

## Authors' contributions

All authors participated in the design of the trial and read and approved the final manuscript.
